# Weight Change as a Predictor of Incidence and Remission of Insulin Resistance

**DOI:** 10.1371/journal.pone.0063690

**Published:** 2013-05-22

**Authors:** Yoosoo Chang, Eunju Sung, Kyung Eun Yun, Hyun-Suk Jung, Chan-Won Kim, Min-Jung Kwon, Sung-Il Cho, Seungho Ryu

**Affiliations:** 1 Center for Cohort Studies, Total Healthcare Center, Kangbuk Samsung Hospital, Sungkyunkwan University, School of Medicine, Seoul, Korea; 2 Department of Occupational and Environmental Medicine, Kangbuk Samsung Hospital, Sungkyunkwan University, School of Medicine, Seoul, Korea; 3 Department of Family Medicine, Kangbuk Samsung Hospital, Sungkyunkwan University, School of Medicine, Seoul, Korea; 4 Department of Laboratory Medicine, Kangbuk Samsung Hospital, Sungkyunkwan University, School of Medicine, Seoul, Korea; 5 Graduate School of Public Health and Institute of Health and Environment, Seoul National University, Seoul, Korea; University of Colorado Denver, United States of America

## Abstract

**Objective:**

The objective of this study was to assess the longitudinal relationship of weight change on incidence and remission of insulin resistance (IR).

**Methods:**

We performed a cohort study in apparently healthy Korean men, 30 to 59 years of age, who underwent a health checkup and were followed annually or biennially between 2002 and 2009. The computer model of homeostasis model assessment, HOMA2-IR, was obtained at each visit, and IR was defined as HOMA2-IR ≥75th percentile.

**Results:**

For IR development, 1,755 of the 6,612 IR-free participants at baseline developed IR (rate 5.1 per 100 person-years) during 34,294.8 person-years of follow-up. The hazard ratios (95% confidence intervals) for incident IR with weight changes of <−0.9 kg, 0.6–2.1 kg and ≥2.2 kg from visit 1 to visit 2 (average 1.8 years) compared to weight change of −0.9–0.5 kg (reference) were 0.78 (0.68–0.90), 1.19 (1.04–1.35) and 1.26 (1.11–1.44), respectively. This association persisted in normal-weight individuals or those without any metabolic syndrome traits and remained significant after introducing weight categories and confounders as time-dependent exposures (*P*-trend <0.001). For IR remission, 903 of 1,696 IR participants had no IR (remission rate 10.3 per 100 person-years) during 8,777.4 person-years of follow-up. IR remission decreased with increasing quartiles of weight change (P-trend <0.001) and this association persisted in normal-weight individuals.

**Conclusions:**

Weight gain was associated with increased IR development and decreased IR remission regardless of baseline BMI status. Preventing weight gain, even in healthy and normal-weight individuals, is an important strategy for reducing IR and its associated consequences.

## Introduction

Insulin resistance (IR), a pathogenic factor for type 2 diabetes [1], is associated with various cardiovascular disease risk factors, including obesity, hypertension and dyslipidemia [2]. Furthermore, IR independently predicts increased occurrence of cardiovascular events [3], [4], cancer [5] and all-cause mortality regardless of metabolic syndrome (MetS) or its individual components [3]. Therefore, identifying risk factors of insulin resistance is of great importance. There are well established associations between obesity and IR [6], but IR is also encountered in non-obese, non-diabetic individuals [7]. However, the multiple mechanisms causing IR in lean individuals are not yet fully understood.

A few experimental studies have shown that weight gain-indueced IR was observed by short term overfeeding within one or several months [8]–[10] and showed the effect of weight gain on insulin resistance in a limited number of non-obese individuals [9]. Previous observational studies have shown that weight gain from age of 20 years to middle age is associated with increased risk of hyperinsulinemia [11], [12]. The association between weight gain and IR was not described among normal-weight individuals. Regarding IR remission, many studies have suggested that weight reduction improves insulin sensitivity and/or diabetes status in selected populations such as those with obesity and/or diabetes [13]–[16]. Far less is known about the relationship between weight change and IR remission in apparently healthy, non-diabetic, and non-obese population, especially in normal-weight individuals.

The homeostasis model assessment (HOMA), a validated method for assessing IR [17], is commonly used in clinical and epidemiological studies, and can be used to assess longitudinal changes in subjects [18]. Recently, HOMA2-IR, the updated computer model, was developed with physiological adjustments for renal glucose loss and peripheral and hepatic glucose resistance, providing a more accurate index [18], [19]. Until now, no longitudinal study has addressed the association between weight change and IR using HOMA2-IR.

This study examined the association between short-term (1- or 2-years, mean 1.8 years) spontaneous weight change over time on the incidence and remission of IR, estimated by HOMA2-IR, in apparently healthy Korean men and whether or not this association persists in normal-weight individuals.

## Subjects and Methods

### Subjects

The study sample included male employees of one of the largest semiconductor companies in Korea and its 13 affiliates [20]–[22]. and were followed annually or biennially between 2002 and 2009. In Korea, the Industrial Safety and Health Law requires that employees participate in annual or biennial health examinations. The potential subjects were all male and ranged from 30 to 59 years of age. All participated in comprehensive health examinations at the Kangbuk Samsung Hospital, Seoul, Korea in 2002 (n = 15,347).

**Figure 1:**
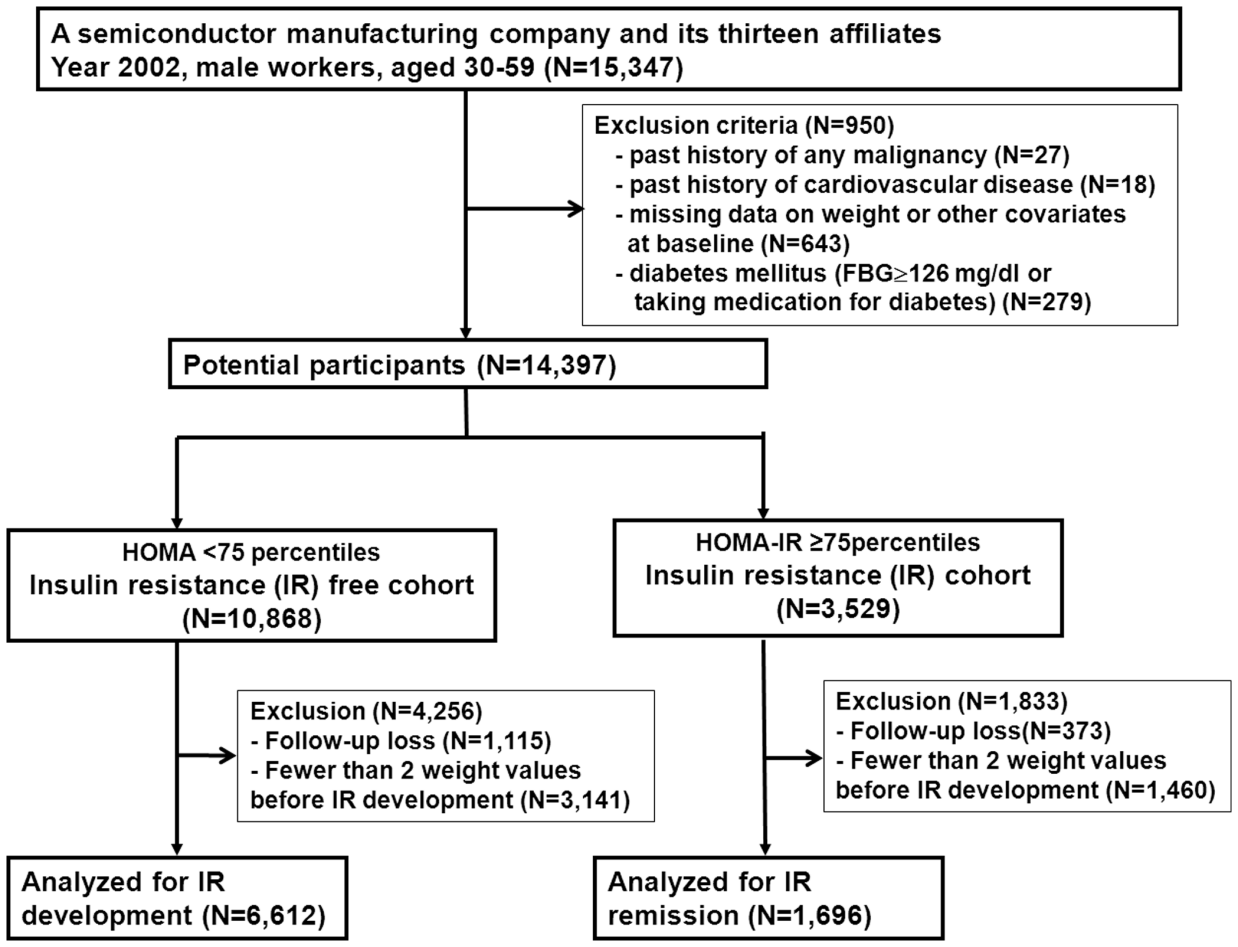
Flow diagram for the selection of study subjects.

For this analysis, we excluded participants with diabetes mellitus, or with factors that could influence insulin resistance (Figure 1): we excluded subjects with a history of malignancy, cardiovascular disease or diabetes mellitus (fasting blood glucose≥126 mg/dl or reported treatment with oral anti-diabetic agents or insulin) and subjects with missing data on weight or other covariates at baseline. The sample size for eligible population was 14,397 at baseline. Among them, 3,529 individuals were classified as having IR defined by HOMA2-IR ≥75 percentiles [23] at baseline and remaining individuals (n = 10,868) comprised IR-free cohort. Furthermore, 1,115 subjects (10.3%) from IR free cohort and 373 subjects (10.6%) from IR cohort were excluded for not attending any follow-up visit after the baseline visit. Finally, 6,612 subjects and 1,696 subjects who had at least two weight measurements recorded prior to IR assessment were included in each analysis for either IR development or IR remission, respectively. Compared to participants of IR free cohort included in the analyses, 4,256 who met either follow-up loss or less than two weight measurements prior to IR assessment and were not included were, on average, 1.0 years younger and had less favorable metabolic profiles at baseline (data not shown). Among the 3,529 eligible participants of the IR cohort at baseline, 1,833 who met either follow-up loss or less than two weight measurements prior to IR assessment and were not included in the analytic cohort were, on average, 2.0 years older, more likely to be a current drinker and had a more favorable metabolic profile at baseline (data not shown).

This study was approved by the Institutional Review Board of Kangbuk Samsung Hospital, which exempted the requirement for informed consent as we only retrospectively accessed data that were de-identified.

### Measurements

Study subjects were examined at the Kangbuk Samsung Hospital Health Screening Center, annually or biennially between January 2002 and December 2009. Data on medical history, health-related behaviors, physical measurements, and serum biochemical measurements were collected at each visit [21]. Self-administered questionnaire data were used to identify current smokers and to assess the weekly frequency of moderate- or vigorous-intensity physical activity. Questions regarding alcohol intake included weekly frequency of alcohol consumption and usual daily amount of consumption. Body weight was measured, while each subject wore light clothing and no shoes, to the nearest 0.1 kilogram using a digital scale. Height was measured to the nearest 0.1 centimeter. Trained nurses measured sitting blood pressure (BP) with standard mercury sphygmomanometers.

Blood samples were taken from the antecubital vein after at least a 10-hour fast. Serum levels of glucose, uric acid, total cholesterol, triglyceride, low-density lipoprotein cholesterol (LDL-C), high-density lipoprotein cholesterol (HDL-C), and liver enzymes were measured using Bayer Reagent Packs (Advia 1650™ Autoanalyzer; Bayer Diagnostics, Leverkusen, Germany). Measurement techniques included the hexokinase method for glucose, and an enzymatic colorimetric assay for serum lipids.

Serum insulin levels were measured with immunoradiometric assays (Biosource, Nivelles, Belgium), with intra- and inter-assay coefficients of variation of 4.7–12.2% and the analytical sensitivity of 1 uIU/ml. IR was determined by the homeostasis model assessment of IR (HOMA2-IR) based on fasting blood glucose and insulin, using the computer-based model, accessed at http://www.dtu.ox.ac.uk/homa. HOMA1-IR was calculated by the formula: HOMA-IR = [glucose (mmol/L) X insulin (uIU/mL)] ÷ 22.5. HOMA2-IR excel sheet limits the concentration ranges of 3–25 mmol/L for glucose and 2.9–57.6 mIU/L for insulin, the total number of results differed between HOMA1-IR and HOMA2-IR. To define individuals as having IR at baseline or at follow-up, we used as cutoff value the 75th percentile [23] of baseline HOMA-IR (1.26 for HOMA2-IR and 2.2 for HOMA1-IR in this study population).

High-sensitivity C-reactive protein (HsCRP) was analyzed by particle-enhanced immunonephelometry with the BNII™ System (Dade Behring, Marburg, Germany) using a lower detection limit of 0.175 mg/L. The clinical laboratory was accredited and participates annually in inspections and surveys by the Korean Association of Quality Assurance for Clinical Laboratories.

Abdominal ultrasounds were performed using a Logic Q700 MR 3.5-MHz transducer (GE, Milwaukee, WI, USA) by 12 experienced radiologists who were unaware of the aims of the study. An ultrasonographic diagnosis of fatty liver was defined as the presence of a diffuse increase of fine echoes in the liver parenchyma compared with the kidney or spleen parenchyma [24].

MetS was defined as the presence of three or more ATP III criteria [25]: 1) abdominal obesity; 2) fasting blood glucose ≥100 mg/dL; 3) triglycerides ≥150 mg/dL; 4) HDL-cholesterol <40 mg/dL and 5) BP≥130/85 mm Hg. Since waist circumference measurements were not available for all subjects, we substituted overall adiposity (i.e., BMI ≥25 kg/m^2^, which has been proposed as a cut-off for the diagnosis of obesity in Asians [26]) for abdominal obesity. Hypertension was defined as use of anti-hypertensive medication or BP≥140/90 mm Hg. Diabetes was defined as a fasting serum glucose ≥126 mg/dL or current use of blood glucose lowering agents.

### Statistical Analyses

The χ^2^-test and one-way ANOVA were used to compare the characteristics of the study participants at baseline according to weight change category. The distribution of continuous variables was evaluated and appropriate transformations were done during analyses, as needed.

Weight changes were calculated for each subject as the differences in weight from visit 2 to baseline (visit 1), from visit 3 to visit 2, from visit 4 to visit 3, from visit 5 to visit 4 and from visit 6 to visit 5. First weight change as ‘visit 2 weight’ minus ‘baseline weight’ was categorized into quartiles based on its distribution. For IR free cohort, we used the following quartiles: <−0.9, −0.9–0.5 (reference), 0.6–2.1 and ≥2.2 kg. A category containing 0 kg was used as a reference group. We considered regular exercise (less than once per week or one or more times per week). BMI was categorized based on criteria for Asian populations [26], [27]: underweight = BMI <18.5 kg/m^2^; normal-weight = BMI of 18.5–23 kg/m^2^; overweight = BMI of 23–25 kg/m^2^ and obesity = BMI ≥25 kg/m^2^.

Incidence density was expressed as the number of cases divided by person-years. The cumulative incidence of 2 year, 4 years and 6 years was expressed by a simple proportion.

Since we knew that metabolic abnormalities had occurred between two visits but did not know the precise time of each metabolic abnormality development, we used a parametric Cox model to take into account this type of interval censoring (*stpm* command in Stata) [28]. In these models, the baseline hazard function was parameterized with restricted cubic splines in log time with four degrees of freedom. We estimated adjusted hazard ratios (aHR) with 95% confidence intervals (CI) for incident IR comparing weight loss and weight gain to weight stable category. For time-dependent analyses, we used a pooled logistic regression model that closely approximates a Cox model when the risk of outcome between intervals is low [29].

In the IR free cohort, follow-up extended from the baseline exam until the development of IR or the last health exam conducted for each participant, whichever occurred first. For incident IR, time-dependent analyses were carried out using the weight and covariates, before IR development. And the incident IR individuals were not transferred to the IR cohort to look at remission rates. For the IR cohort, likewise, study participants were considered to be at risk from the baseline exam until IR remission or the last health exam conducted for each participant, whichever occurred first. For remission of IR, time-dependent analyses were carried out using the weight and covariates, before IR remission. The models were initially adjusted for age, and then for BMI, smoking, alcohol intake and exercise. To determine linear trends of risk, the number of categories or quartiles was used as a continuous variable and tested on each model. We assessed the proportional hazards assumption by examining graphs of estimated log(−log) survival. Subgroup analyses was conducted according to baseline BMI status, MetS and smoking status (current smoker vs noncurrent smoker): and multiplicative interaction terms were used to examine if the association of weight change category with the risk of developing IR and IR remission varied by subgroups. Sensitivity analyses were done using HOMA1-IR, instead of HOMA2-IR.

Statistical analyses were performed using STATA version 11.2 (StataCorp LP, College Station, TX, USA). All P values were 2-tailed, and statistical significance was set at P<0.05.

## Results

At baseline, the mean (standard deviation) age and BMI of study participants in the IR-free cohort was 37.6 years (5.0) and 23.2 kg/m^2^ (2.5), respectively (Table 1). Study participants in the IR cohort were more likely to be obese and have fatty liver, metabolic syndrome and worse profiles of metabolic parameters (Tables 1 and 2). In both the IR-free and IR cohorts, BMI, glucose, BP, triglyceride, liver enzymes and CRP were associated inversely with weight change, whereas current smoking was associated positively. The proportions of hypertension, MetS, fatty liver and obesity were also inversely associated with weight change. Likewise, in both the cohorts, weight gain was more likely to occur in individuals with better metabolic profile than those with higher BMI and poorer metabolic profile.

**Table 1:** Baseline characteristics of study participants in insulin resistance free cohort by weight change category.

	Overall	Weight change (kg) quartiles over the 1.8 years from visit 1 to visit 2				*P*-trend
		Q1 (<–0.9)	Q2 (–0.9∼0.5)	Q3 (0.6∼2.0)	Q4 (≥2.1)	
Number	6,612	1,662	1,707	1,592	1,651	
Age (years)^*^	37.6 (5.0)	37.4 (4.9)	38.1 (5.0)	38.2 (5.1)	37.0 (4.9)	0.12
BMI (kg/m^2^)^*^	23.2 (2.5)	23.9 (2.5)	23.2 (2.5)	23.1 (2.4)	22.6 (2.3)	<0.001
BMI categories (kg/m^2^) (%)						
Underweight (<18.5)	2.3	1.2	2.9	2.1	2.9	0.01
Normal-weight (18.5–22.9)	43.0	32.6	42.2	44.1	53.4	<0.001
Overweight (23.0–24.9)	30.0	30.6	31.4	30.8	27.1	0.03
Obesity (≥25.0)	24.7	35.6	23.6	23.0	16.5	<0.001
Current smoker (%)	45.8	41.9	45.4	46.3	49.7	<0.001
Alcohol use (%)‡	16.2	15.9	16.8	16.3	15.8	0.81
Regular exercise (%)§	52.0	52.7	51.1	52.4	51.8	0.79
Hypertension (%)	14.0	16.7	13.8	13.9	11.4	<0.001
Metabolic syndrome (%)	9.8	14.7	10.0	8.4	6.1	<0.001
Fatty liver (%)	19.4	25.3	20.7	18.4	13.1	<0.001
Systolic BP (mmHg)^*^	114.5 (12.4)	115.7 (12.9)	114.4 (12.4)	114.2 (12.3)	113.5 (12.0)	<0.001
Diastolic BP (mmHg)^*^	74.3 (10.1)	75.3 (10.5)	74.4 (9.9)	74.4 (10.0)	73.3 (9.7)	<0.001
Glucose (mg/dl)*	89.8 (9.1)	91.1 (9.3)	90.3 (9.0)	89.6 (9.0)	88.4 (8.9)	<0.001
Total cholesterol	199.0 (33.7)	201.8 (34.8)	199.6 (33.2)	199.3 (33.4)	195.3 (33.2)	<0.001
LDL-C (mg/dl)*	118.4 (28.7)	120.4 (30.1)	118.4 (28.5)	118.7 (28.0)	116.2 (28.1)	0.001
HDL-C (mg/dl )*	53.5 (11.5)	52.5 (11.0)	53.7 (11.8)	53.5 (11.7)	54.3 (11.5)	<0.001
Uric acid (mg/dl)*	5.90 (1.11)	5.99 (1.16)	5.92 (1.09)	5.85 (1.12)	5.85 (1.08)	<0.001
Triglycerides (mg/dl)†	117 (86–163)	126 (91–175)	119 (87–167)	116 (86–163)	108 (80–149)	<0.001
Alanine aminotrasferase (U/l)†	23.0 (17.0–31.0)	25.0 (18.0–35.0)	23.0 (18.0–32.0)	23.0 (17.0–31.0)	22.0 (17.0–29.0)	<0.001
Aspartate aminotrasferase (U/l)†	23.0 (20.0–27.0)	23.0 (20.0–28.0)	23.0 (20.0–27.0)	23.0 (20.0–27.0)	22.0 (19.0–26.0)	<0.001
γ-glutamyltransferase (U/l)†	23.0 (16.0–35.0)	24.0 (17.0–37.0)	23.0 (17.0–35.0)	23.0 (16.5–36.0)	21.0 (16.0–31.0)	<0.001
hsCRP (mg/l)†	0.40 (0.20–0.90)	0.50 (0.30–0.90)	0.40 (0.20–0.80)	0.40 (0.20–0.90)	0.40 (0.20–0.90)	0.01
Insulin (pmol l−1)	6.20 (5.07–7.57)	6.52 (5.32–7.80)	6.22 (5.01–7.65)	6.21 (5.05–7.54)	5.87 (4.92–7.25)	<0.001
HOMA2-IR	0.81 (0.66–0.99)	0.85 (0.70–1.02)	0.81 (0.65–1.00)	0.81 (0.65–0.99)	0.76 (0.64–0.94)	<0.001

Data are ^*^mean (standard deviation),

† median (interquartile range) or percentage.

**Abbreviations:** BMI, body mass index; HDL-C, high-density lipoprotein-cholesterol; hsCRP, high sensitivity C-reactive protein; HOMA-IR, homeostasis model assessment of insulin resistance; LDL-C, low-density lipoprotein-cholesterol.

‡ ≥20 g of ethanol per day.

§ ≥1 time/week.

**Table 2:** Baseline characteristics of study participants in insulin resistance cohort by weight change category.

	Overall	Weight change (kg) quartiles over the 1.8 years from visit 1 to visit 2						*P*-trend
		I (<–1.1)	II (–1.0∼0.6)		III (0.6∼2.2)		IV (≥2.2)	
Number	1,696	438		424		410	424	
Age (years)^*^	36.2 (4.5)	36.0 (4.1)		36.8 (4.5)		36.3 (4.7)	35.8 (4.5)	0.40
BMI (kg/m^2^)^*^	26.6 (2.7)	27.2 (2.6)		26.5 (2.5)		26.5 (2.6)	26.1 (2.7)	<0.001
BMI categories (kg/m^2^) (%)								
Underweight (<18.5)	0.0	0.0		0.0		0.0	0.0	–
Normal-weight (18.5–22.9)	7.3	3.9		5.7		8.3	11.6	<0.001
Overweight (23.0–24.9)	19.5	16.0		21.2		19.3	21.5	0.09
Obesity (≥25.0)	73.2	80.1		73.1		72.4	67.0	<0.001
Current smoker (%)	44.6	41.6		41.5		45.1	50.2	0.01
Alcohol intake (%)‡	15.6	15.1		17.0		14.6	15.8	0.99
Regular exercise (%)§	45.5	46.4		47.2		42.7	45.5	0.52
Hypertension (%)	25.4	29.5		26.2		24.6	21.2	0.01
Metabolic syndrome (%)	41.9	45.4		46.7		42.2	33.0	<0.001
Fatty liver (%)	66.3	73.5		67.2		69.0	55.4	<0.001
Systolic BP (mmHg)^*^	120.3 (14.4)	121.3 (14.4)		120.3 (14.3)		120.0 (14.5)	119.7 (14.1)	0.09
Diastolic BP (mmHg)^*^	78.8 (11.4)	79.6 (11.7)		79.2 (11.6)		78.5 (10.8)	77.8 (11.2)	0.01
Glucose (mg/dl)^*^	95.9 (9.3)	97.3 (9.1)		95.9 (9.6)		96.1 (9.2)	94.1 (9.0)	<0.001
Total cholesterol	214.2 (36.2)	217.1 (37.9)		215.2 (35.7)		211.6 (33.9)	212.7 (36.9)	0.03
LDL-C (mg/dl)^*^	128.0 (31.2)	129.0 (31.1)		127.6 (31.7)		126.3 (30.3)	129.1 (31.7)	0.86
HDL-C (mg/dl )^*^	47.3 (9.7)	47.3 (9.5)		47.4 (9.7)		46.8 (9.4)	47.8 (10.1)	0.60
Uric acid (mg/dl)^*^	6.54 (1.22)	6.62 (1.27)		6.49 (1.23)		6.53 (1.19)	6.51 (1.20)	0.29
Triglycerides (mg/dl)†	177 (128–249.5)	183 (130–259)		186 (139–260)		183 (129–252)	159.5 (122–221)	0.001
Alanine aminotrasferase (U/l)†	42.0 (29.0–64.0)	44.0 (31.0–69.0)		44.0 (32.0–66.0)		43.0 (30.0–65.0)	37.0 (26.0–57.0)	<0.001
Aspartate aminotrasferase (U/l)†	28.0 (23.0–36.0)	29.0 (23.0–38.0)		29.0 (24.0–37.0)		28.0 (24.0–36.0)	27.0 (22.0–34.0)	<0.001
γ-glutamyltransferase (U/l)†	39.0 (26.0–61.0)	41.0 (28.0–67.0)		41.0 (28.0–63.5)		39.0 (26.0–62.0)	36.0 (24.0–55.0)	<0.001
hsCRP (mg/l)†	0.80 (0.50–1.50)	0.90 (0.50–1.70)		0.80 (0.50–1.50)		0.80 (0.40–1.40)	0.80 (0.40–1.50)	0.02
Insulin (pmol/l)	11.06 (10.30–12.60)	11.32 (10.38–13.06)		10.95 (10.29–12.10)		11.03 (10.26–12.62)	11.05 (10.26–12.69)	0.20
HOMA2-IR	1.45 (1.34–1.64)	1.48 (1.36–1.71)		1.43 (1.34–1.59)		1.44 (1.34–1.63)	1.44 (1.33–1.65)	0.06

Data are ^*^mean (standard deviation),

† median (interquartile range) or percentage.

**Abbreviations:** BMI, body mass index; HDL-C, high-density lipoprotein-cholesterol; hsCRP, high sensitivity C-reactive protein; HOMA-IR, homeostasis model assessment of insulin resistance; LDL-C, low-density lipoprotein-cholesterol.

‡ ≥20 g of ethanol per day.

§ ≥1 time/week.

During 34,294.8 person-years of follow-up, 1,755 participants developed IR (incidence rate 5.1 per 100 person-years), whereas during 8,777.4 person-years of follow-up, 903 participants were in remission of IR (remission rate 10.3 per 100 person-years) (Table 3). In both the age-adjusted and multivariate-adjusted models, higher baseline BMI categories predicted the incidence of IR in a graded and dose-response manner, and IR remission decreased with increasing BMI categories (both *P*-trend <0.001). Obesity was associated with a significantly increased risk of IR (aHR, 2.10; 95% CI, 1.87–2.36) and significantly decreased remission of IR (aHR, 0.66; 95% CI, 0.52–0.84). These associations persisted after introducing BMI categories and confounding factors as time-dependent exposures (*P*-trend <0.001).

**Table 3:** Development of insulin resistance (IR) and the remission of IR according to baseline body mass index category.

BMI (kg m^−2^) category	Person-years	Incident case	Incidence density (100 person-year)	Cumulative incidence (%)			Age-adjusted HR (95% CI)	Multivariate HR* (95% CI)	HR (95% CI)‡ in the model using time-dependent variables
				2 year	4 year	6 year			
**Development of IR from IR free cohort**									
<18.5	886.1	10	1.1	0.7	3.3	4.6	0.40 (0.21–0.75)	0.40 (0.21–0.74)	0.13 (0.02–0.95)
18.5–22.9	15,313.9	543	3.5	3.4	11.7	15.7	1.00 (reference)	1.00 (reference)	1.00 (reference)
23.0–24.9	10,171.2	579	5.7	6.3	19.3	24.4	1.53 (1.36–1.72)	1.54 (1.37–1.73)	1.61 (1.35–1.92)
≥25.0	7,923.6	623	7.9	9.1	25.0	31.9	2.07 (1.85–2.33)	2.10 (1.87–2.36)	2.42 (2.04–2.87)
P for trend							<0.001	<0.001	<0.001
**Remission of IR from IR cohort**									
<18.5	0	0	0	0	0	0	-	-	-
18.5–22.9	661.8	79	11.9	1.6	13.7	41.9	1.00 (reference)	1.00 (reference)	1.00 (reference)
23.0–24.9	1,720.9	201	11.7	3.9	14.5	39.1	0.89 (0.68–1.16)	0.88 (0.68–1.15)	0.83 (0.45–1.54)
≥25.0	6,394.8	623	9.7	4.1	17.6	36.9	0.66 (0.52–0.84)	0.66 (0.52–0.84)	0.45 (0.26–0.77)
P for trend							<0.001	<0.001	<0.001

* estimated from Parametric Cox models adjusted for age, smoking status, alcohol intake, and regular exercise at baseline;

‡ estimated from a pooled logistic regression models with BMI as a time-dependent categorical variable adjusted for other covariates (baseline age and current smoker, current alcohol use, and regular exercise over time as time-dependent variables).

Abbreviations: BMI, body mass index; CI, confidence intervals; IR, insulin resistance; HR, hazard ratio.

Table 4 shows the risk of developing IR and IR remission according to weight change categories. The median time (interquartile ranges) of the interval between visit 1 and visit 2 among study subjects was 1.8 years (1.0–2.1). In both the age-adjusted and multivariate-adjusted models, the risk for IR increased with increasing quartiles of weight change (*P*-trend <0.001). The aHRs (95% CIs) for IR in the weight change <−0.9 kg, 0.6–2.1 kg and ≥2.2 kg compared to weight change of −0.9–0.5 kg (reference) were 0.78 (0.68–0.90), 1.19 (1.04–1.35) and 1.26 (1.11–1.44), respectively. This association persisted after introducing weight categories and confounding factors as time-dependent exposures (*P*-trend <0.001). And in both the age-adjusted and multivariate-adjusted models, the number of subjects with IR remission decreased with increasing quartiles of weight change (*P*-trend <0.001). Subjects who gained ≥2.2 kg had a significantly decreased remission of IR (aHR, 0.81; 95% CI, 0.67–0.98). However, the association did not remain significant after introducing weight change and confounding factors as time-dependent exposures.

**Table 4:** Development of insulin resistance (IR) and the remission of IR according to weight change (kg) over 1.8 years, between visit 1 and visit 2.

Quartiles of weight change (kg) over 1.8 years	Person-years	Incident case	Incidence density (100 person-year)	Age-adjusted HR (95% CI)	Multivariate HR* (95% CI)	HR (95% CI)‡ in the model using time-dependent variables
**Development of IR from IR free cohort**						
Q1 (<–0.9)	8,741.6	373	4.3	0.89 (0.78–1.02)	0.78 (0.68–0.90)	0.80 (0.66–0.98)
Q2 (–0.9∼0.5)	8,756.0	449	5.1	1.00 (reference)	1.00 (reference)	1.00 (reference)
Q3 (0.6∼2.1)	8,105.8	481	5.9	1.16 (1.02–1.32)	1.19 (1.04–1.35)	1.20 (0.99–1.46)
Q4 (≥2.2)	8,691.3	452	5.2	1.14 (1.00–1.30)	1.26 (1.11–1.44)	1.34 (1.06–1.68)
P for trend				<0.001	<0.001	<0.001
**Remission of IR from IR cohort**						
Q1 (<–1.1)	2,287.7	253	11.1	1.08 (0.91–1.30)	1.15 (0.96–1.37)	1.45 (0.97–2.16)
Q2 (–1.1∼0.5)	2,191.3	235	10.7	1.00 (reference)	1.00 (reference)	1.00 (reference)
Q3 (0.6∼2.1)	2,062.4	210	10.2	0.96 (0.80–1.16)	0.96 (0.79–1.15)	1.36 (0.87–2.11)
Q4 (≥2.2)	2,236.0	205	9.2	0.84 (0.70–1.02)	0.81 (0.67–0.98)	1.19 (0.75–1.91)
P for trend				0.01	<0.001	0.637

* estimated from Parametric Cox models adjusted for age, BMI, smoking status, alcohol intake, and regular exercise at baseline.

‡ estimated from a pooled logistic regression models with weight change as a time-dependent categorical variable adjusted for other covariates (baseline age and current smoker, current alcohol use, regular exercise and BMI over time as time-dependent variables).

Weight change was categorized into quartiles based on the distribution of weight changes of each group. A category containing 0 kg was used as a reference group.

Abbreviations: BMI, body mass index; CI, confidence intervals; IR, insulin resistance; HR, hazard ratio.

The risk of developing IR and IR remission according to weight change categories were similar across the subgroups of study participants with no statistically significant interactions with BMI (<25, ≥25 kg/m^2^), MetS (yes, no) and smoking status (current smoker vs noncurrent smoker) (Table 5). In subgroup analyses, the risk for IR increased with increasing quartiles of weight change, even in normal-weight subjects or subjects without any MetS traits. This association was observed regardless of smoking status. In overweight and obese subjects, weight loss (lowest quartile of weight change) was associated with a significantly decreased risk of developing IR compared to stable weight (reference). This association was observed regardless of MetS. Weight gain (the highest quartile of weight change) was associated with a increased risk of developing IR in normal-weight subjects and subjects without MetS or any MetS traits. All these associations between weight loss, weight gain and incident IR were observed regardless of smoking status.

**Table 5:** Development and remission of insulin resistance according to weight change in clinically relevant subgroups.

	Quartiles of weight change (kg) over the 1.8 years from visit 1 to visit 2						*P*-trend
Subgroups	Q1		Q2	Q3		Q4	
**Development of IR among IR free cohort**							
18.5≤ BMI <23.0 kg/m^2^ (n = 2,845)		−8.9∼−0.5	−0.4∼0.9		1.0∼2.5	2.6∼13.6	
Multivariate HR* (95% CI)		0.79 (0.61–1.02)	1.00 (reference)		1.33 (1.06–1.69)	1.27 (1.00–1.61)	<0.001
23.0≤ BMI <25.0 kg/m^2^ (n = 1,982)		−9.9∼−1.0	−0.9∼0.4		0.5∼2.0	2.1∼9.4	
Multivariate HR* (95% CI)		0.66 (0.51–0.84)	1.00 (reference)		0.99 (0.79–1.23)	1.22 (0.98–1.52)	<0.001
BMI ≥25.0 kg/m^2^ (n = 1,633)		−15.3∼−1.8	−1.8∼−0.2		−0.1∼1.4	1.5∼13.1	
Multivariate HR* (95% CI)		0.60 (0.47–0.76)	0.79 (0.64–0.98)		1.00 (reference)	0.93 (0.75–1.15)	<0.001
Without MetS (n = 5,963)		−15.3∼−0.9	−0.8∼0.5		0.6∼2.2	2.3∼13.6	
Multivariate HR* (95% CI)		0.74 (0.64–0.86)	1.00 (reference)		1.09 (0.95–1.25)	1.22 (1.06–1.40)	<0.001
With MetS (n = 649)		−12.5∼−1.8	−1.7∼−0.2		−0.1∼1.1	1.2∼13.1	
Multivariate HR* (95% CI)		0.58 (0.40–0.84)	0.82 (0.58–1.15)		1.00 (reference)	0.94 (0.67–1.31)	0.01
Without any MetS traits (n = 2,716)		−9.9∼−0.6	−0.5∼0.8		0.9∼2.5	2.6∼13.6	
Multivariate HR* (95% CI)		0.72 (0.56–0.94)	1.00 (reference)		1.06 (0.84–1.34)	1.39 (1.10–1.76)	<0.001
Current smoker (n = 3,029)		−12.5∼−0.8	−0.7∼0.5		0.6∼2.2	2.3∼13.6	
Multivariate HR* (95% CI)		0.79 (0.64–0.97)	1.00 (reference)		1.05 (0.86–1.28)	1.29 (1.06–1.57)	<0.001
Non-smoker or ex-smoker (n = 3583)		−15.3∼−1.1	−1.0∼0.3		0.4∼2.0	2.1∼10.9	
Multivariate HR* (95% CI)		0.78 (0.65–0.94)	1.00 (reference)		1.20 (1.01–1.43)	1.26 (1.05–1.50)	<0.001
**Remission of IR among IR cohort**							
18.5≤ BMI <23.0 kg/m^2^ (n = 124)		−7.0∼0.2	0.3∼1.6		1.7∼3.2	3.2∼11.9	
Multivariate HR* (95% CI)		1.00 (reference)	0.48 (0.25–0.91)		0.69 (0.35–1.36)	0.35 (0.17–0.71)	0.014
23.0≤ BMI <25.0 kg/m^2^ (n = 330)		−10.3∼−0.7	−0.6∼0.8		0.9∼2.4	2.5∼11.6	
Multivariate HR* (95% CI)		1.20 (0.80–1.79)	1.00 (reference)		1.09 (0.72–1.65)	0.92 (0.61–1.38)	0.26
BMI ≥25.0 kg/m^2^ (n = 1,242)		−14.5∼−1.3	−1.3∼0.5		0.6∼2.0	2.1∼21.3	
Multivariate HR* (95% CI)		1.07 (0.87–1.33)	1.00 (reference)		0.91 (0.73–1.14)	0.79 (0.63–0.98)	0.005
Without MetS (n = 986)		−14.5∼−1.0	−0.9∼0.8		0.9∼2.6	2.6∼21.3	
Multivariate HR* (95% CI)		1.15 (0.92–1.44)	1.00 (reference)		0.95 (0.75∼1.20)	0.76 (0.60–0.97)	0.001
With MetS (n = 710)		−12.7∼−1.3	−1.3∼0.3		0.4∼1.9	1.9∼8.7	
Multivariate HR* (95% CI)		1.06 (0.79–1.43)	1.00 (reference)		0.93 (0.69∼1.26)	0.81 (0.60–1.10)	0.074
Current smoker (n = 756)		−14.5∼−1.0	−0.9∼0.8		0.9∼2.5	2.6∼21.3	
Multivariate HR* (95% CI)		1.21 (0.91–1.59)	1.00 (reference)		1.15 (0.87∼1.51)	0.83 (0.62–1.12)	0.038
Non-smoker or ex-smoker (n = 940)		−12.3∼−1.2	−1.2∼0.5		0.6∼2.0	2.1∼11.7	
Multivariate HR* (95% CI)		1.14 (0.90–1.45)	1.00 (reference)		0.94 (0.73∼1.21)	0.83 (0.65–1.07)	0.013

Note: The risk of developing IR and IR remission according to weight change categories were similar across the subgroups of study participants with no statistically significant interactions with BMI (<25, ≥25 kg/m^2^), MetS (yes, no) and smoking status (current smoker vs noncurrent smoker).

* estimated from Parametric Cox models adjusted for age, BMI, smoking status, alcohol use, and regular exercise at baseline. Weight change was categorized into quartiles based on the distribution of weight changes of each subgroup. A category containing 0 kg was used as a reference group.

Abbreviations: BMI, body mass index; CI, confidence intervals; HR, hazard ratio; IR, insulin resistance; MetS, metabolic syndrome.

IR remission decreased with increasing quartiles of weight change in normal-weight, obese subjects, or subjects without MetS (Table 5). Weight loss (lowest quartile of weigt change) was associated with statistically insignificant increase in IR remission.

In sensitivity analyses using HOMA1-IR instead of HOMA2-IR (Tables S1 and S2), these analyses qualitatively did not change any of the observed associations.

## Discussion

The present study demonstrates that weight gain over a short period of time (average 1.8 years) was associated with increased IR development and decreased IR remission. These associations were evident in normal-weight individuals and remained significant after introducing weight categories and confounding factors as time-dependent exposures.

Previous experimental studies demonstrated the influence that overfeeding-induced weight gain had on the development of IR within a short period of one or several months in small sample (10 to 40 individuals) [9], [10] with no reference group for comparison. We showed that weight gain increased on the incidence of IR compared with weight stable category in a large population, and spontaneous weight gain in our study may mirror the natural trajectories of weight gain in the general population. Interestingly, weight gain in this study was more likely to occur in healthier men than in men with higher BMI and poorer metabolic profile. Nonetheless, weight gain over a relatively short term was associated with increased risk of new onset IR even in individuals with healthy weight, suggesting that healthy lifestyle messages to prevent weight gain need to be getting through to everyone, not just the obese.

Similiarly, IR remission decreased with increasing quartiles of weight change, even in normal-weight individuals, but the association did not remain significant after introducing weight change and confounding factors as time-dependent exposures, which could suggest that weight gain at baseline appears to affect IR remission more than recent weight gain.

Weight loss was associated with decreased incidence of IR and statistically insignificant increase in IR remission. The protective effect of weight loss on IR incidence is evident especially in overweight and obese individuals or those with MetS. Like previous studies that identified obesity as a central feature of insulin resistance syndrome [30], [31], we found that higher BMI at baseline predicted increased development of IR, but in obese individuals, weight loss was associated with decreased risk of IR onset. In our study, more weight loss and less weight gain in individuals with poorer metabolic profile at baseline could have been due to strong personal motivation and active intervention in these individuals. As a standard of practice, all participants were encouraged to implement a healthy lifestyle but those with a less favorable metabolic profile were likely to be more actively encouraged by the examining physician to implement lifestyle changes. However, after adjusting for smoking status, alcohol use and regular exercise as time-dependent exposures, this association between weight loss and IR incidence remained significant. Regarding the effects of weight loss on IR remission, previous studies showed that weight reduction improved insulin resistance and even resolved diabetes in obese or diabetic patients [13]–[15], [32], [33]. The insignificant benefit of weight loss seen in our study may be due to an insufficient number of obese individuals with weight loss, less severity of obesity (only 2.8% of study population had BMI of ≥30 kg/m^2^) or insufficient degree of weight loss required to improve IR. Adiposity was not directly assessed by more accurate methods, such as dual-energy X-ray absorptiometry. We were unable to differentiate whether weight loss involved fat or lean body mass or between intentional and unintentional weight loss, and this would possibly lead to an underestimate of the beneficial effect of intentional weight loss on IR remission.

Regarding the underlying mechanism between weight change and IR development and remission, though not fully understood, adipose tissue is increasingly recognized as an active endocrine organ by releasing adipocytokines [34], of which some have important roles in the pathogenesis of IR [34]–[36]. Increased fat mass leads to the dysregulation of adipose tissue functions, including oversecretion of deleterious adipokines and hyposecretion of beneficial ones, such as adiponectin [37]. With weight loss or gain result in changes in circulating levels of adipocytokines [13], [38]–[42], which can influence insulin sensitivity.

There were several limitations in the study. Firstly, for estimation of HOMA-IR, we used a single measurement at each visit. In practice, a single sample is often taken and is acknowledged to be adequate for assessing IR at the population level [18]. If the misclassification of IR using single measurement were not associated with either weight status or weight change, this would bias results towards the null, possibly leading to an underestimate of the association. Secondly, we were unable to obtain dietary information as a possible confounder even though IR or weight status can be affected by dietary habits [43]. Lastly, findings from this study of apparently healthy Korean men may not be generalizable to other ethnic populations or to women. However, our study has several strengths. We were able to perform all analyses based on measured values of weight, blood glucose and insulin when taking into account other potential confounders. A large sample size made it possible to show a significant association between weight change and IR development and remission in various subgroups. Weight gain in normal-weight individuals, was related to increased IR incidence and decreased remission of IR.

In conclusion, weight gain during adulthood adversely affected both the incidence and remission of IR regardless of baseline BMI status. Therefore, strategies to prevent weight gain should be directed to the whole spectrum of non-obese to obese men, expecting to contribute to reduce IR and its associated consequences.

## Supporting Information

Table S1 **The development of insulin resistance (IR) and the remission of IR using HOMA1-IR according to baseline body mass index category.**
(DOCX)

Table S2 **The development of insulin resistance (IR) and the remission of IR using HOMA1-IR according to weight change (kg) over 1.8 years, between visit 1 and visit 2.**
(DOC)
