# Supplementary material for: Weight Change as a Predictor of Incidence and Remission of Insulin Resistance
Source: PLoS One. 2013 May 22;8(5):e63690. doi: 10.1371/journal.pone.0063690 (PMC3661661; doi:10.1371/journal.pone.0063690)
Supplement: Table S1 — The development of insulin resistance (IR) and the remission of IR using HOMA1-IR according to baseline body mass index category. (DOCX) [file pone.0063690.s001.docx]

**Table S1. The development of insulin resistance (IR) and the remission of IR using HOMA1-IR according to baseline body mass index category**

| BMI (kg m^-2^)  category | Person-years | Incident  case | Incidence Density (100 person-year) | Cumulative incidence (%) | | | Age-adjusted HR  (95% CI) | Multivariate HR^*^  (95% CI) | HR (95% CI)^‡^  in the model using time-dependent variables |
| --- | --- | --- | --- | --- | --- | --- | --- | --- | --- |
|  |  |  |  | 2 year | 4 year | 6 year |  |  |  |
| **Development of IR from IR free cohort** | | |  |  |  |  |  |  |  |
| <18.5 | 887.2 | 18 | 2.0 | 0.7 | 6.6 | 10.5 | 0.45 (0.28-0.70) | 0.44 (0.28-0.71) | 0.21 (0.05-0.88) |
| 18.5-22.9 | 14,831.4 | 756 | 5.1 | 2.9 | 15.8 | 24.3 | 1.00 (reference) | 1.00 (reference) | 1.00 (reference) |
| 23.0-24.9 | 9,391.9 | 784 | 8.3 | 6.4 | 26.8 | 37.0 | 1.62 (1.46-1.79) | 1.63 (1.48-1.80) | 1.79 (1.51-2.11) |
| ≥25.0 | 7,039.7 | 810 | 11.5 | 8.8 | 35.6 | 47.5 | 2.25 (2.04-2.49) | 2.28 (2.06-2.52) | 2.57 (2.18-3.04) |
| P for trend |  |  |  |  |  |  | <0.001 | <0.001 | <0.001 |
| **Remission of IR from IR cohort** | | |  |  |  |  |  |  |  |
| <18.5 | 0 | 0 | 0 | 0 | 0 | 0 | - | - | - |
| 18.5-22.9 | 672.0 | 101 | 15.0 | 0.7 | 27.0 | 62.0 | 1.00 (reference) | 1.00 (reference) | 1.00 (reference) |
| 23.0-24.9 | 1,772.6 | 220 | 12.4 | 2.3 | 21.7 | 51.3 | 0.74 (0.58-0.94) | 0.75 (0.59-0.95) | 0.92 (0.53-1.58) |
| ≥25.0 | 7,008.1 | 627 | 8.9 | 1.4 | 16.2 | 37.2 | 0.48 (0.39-0.59) | 0.49 (0.39-0.60) | 0.49 (0. 30-0.80) |
| P for trend |  |  |  |  |  |  | <0.001 | <0.001 | <0.001 |

^*^estimated from Parametric Cox models adjusted for age, smoking status, alcohol intake, and regular exercise at baseline

^‡^estimated from a pooled logistic regression models with BMI as a time-dependent categorical variable adjusted for other covariates (baseline age and current smoker, current alcohol use, and regular exercise over time as time-dependent variables)

Abbreviations: BMI, body mass index; CI, confidence intervals; IR, insulin resistance; HR, hazard ratio
