# Supplementary material for: Weight Change as a Predictor of Incidence and Remission of Insulin Resistance
Source: PLoS One. 2013 May 22;8(5):e63690. doi: 10.1371/journal.pone.0063690 (PMC3661661; doi:10.1371/journal.pone.0063690)
Supplement: Table S2 — The development of insulin resistance (IR) and the remission of IR using HOMA1-IR according to weight change (kg) over 1.8 years, between visit 1 and visit 2. (DOC) [file pone.0063690.s002.doc]

**Table S2. The development of insulin resistance (IR) and the remission of IR using HOMA1-IR according to weight change** (kg) over 1.8 years, between visit 1 and visit 2

| Quartiles of weight change (kg) over 1.8 years | Person-years | Incident  case | Incidence Density (100 person-year) | Age-adjusted HR  (95% CI) | Multivariate HR^*^  (95% CI) | HR (95% CI)^‡^  in the model using time-dependent variables |
| --- | --- | --- | --- | --- | --- | --- |
|
| **Development of IR from IR free cohort** | | |  |  |  |  |
| I (<–0.9) | 8,363.3 | 523 | 6.3 | 0.83 (0.74-0.93) | 0.72 (0.64-0.81) | 0.78 (0.65-0.94) |
| II (–0.9~0.5) | 8,127.2 | 640 | 7.9 | 1.00 (reference) | 1.00 (reference) | 1.00 (reference) |
| III (0.6~2.1) | 7,567.6 | 604 | 8.0 | 1.03 (0.92-1.15) | 1.06 (0.95-1.18) | 1.25 (1.05-1.49) |
| IV (≥2.2) | 8,092.1 | 601 | 7.4 | 1.01 (0.90-1.13) | 1.12 (1.01-1.25) | 1.43 (1.15-1.78) |
| P for trend |  |  |  | 0.001 | <0.001 | <0.001 |
| **Remission of IR from IR cohort** | | |  |  |  |  |
| I (<–1.3) | 2,348.1 | 275 | 11.7 | 1.09 (0.92-1.30) | 1.19 (1.00-1.41) | 1.15 (0.79-1.69) |
| II (–1.3~0.4) | 2,389.7 | 264 | 11.0 | 1.00 (reference) | 1.00 (reference) | 1.00 (reference) |
| III (0.5~2.0) | 2,353.8 | 214 | 9.1 | 0.80 (0.67-0.96) | 0.80 (0.67-0.96) | 1.29 (0.87-1.91) |
| IV (≥2.1) | 2,361.1 | 195 | 8.3 | 0.71 (0.59-0.85) | 0.69 (0.57-0.83) | 1.09 (0.71-1.69) |
| P for trend |  |  |  | <0.001 | <0.001 | 0.832 |

^*^estimated from Parametric Cox models adjusted for age, BMI, smoking status, alcohol intake, and regular exercise at baseline

^‡^estimated from a pooled logistic regression models with weight change as a time-dependent categorical variable adjusted for other covariates (baseline age and current smoker, current alcohol use, regular exercise and BMI over time as time-dependent variables)

Weight change was categorized into quartiles based on the distribution of weight changes of each group. A category containing 0 kg was used as a reference group.

Abbreviations: BMI, body mass index; CI, confidence intervals; IR, insulin resistance; HR, hazard ratio
